# Acute Systemic Inflammatory Response Alters Transcription Profile of Genes Related to Immune Response and Ca^2+^ Homeostasis in Hippocampus; Relevance to Neurodegenerative Disorders

**DOI:** 10.3390/ijms21217838

**Published:** 2020-10-22

**Authors:** Grzegorz A. Czapski, Yuhai Zhao, Walter J. Lukiw, Joanna B. Strosznajder

**Affiliations:** 1 Department of Cellular Signalling, Mossakowski Medical Research Centre Polish Academy of Sciences, Pawińskiego 5, 02-106 Warsaw, Poland; 2 LSU Neuroscience Center, Louisiana State University Health Science Center (LSU-HSC), New Orleans, LA 70112, USA; yzhao4@lsuhsc.edu (Y.Z.); wlukiw@lsuhsc.edu (W.J.L.); 3 Department of Cell Biology and Anatomy, LSU-HSC, New Orleans, LA 70112, USA; 4 Department of Ophthalmology, LSU-HSC, New Orleans, LA 70112, USA; 5 Department of Neurology, LSU-HSC, New Orleans, LA 70112, USA

**Keywords:** lipopolysaccharide, systemic inflammation, hippocampus, neuroinflammation, neurodegeneration, microarray

## Abstract

Acute systemic inflammatory response (SIR) triggers an alteration in the transcription of brain genes related to neuroinflammation, oxidative stress and cells death. These changes are also characteristic for Alzheimer’s disease (AD) neuropathology. Our aim was to evaluate gene expression patterns in the mouse hippocampus (MH) by using microarray technology 12 and 96 h after SIR evoked by lipopolysaccharide (LPS). The results were compared with microarray analysis of human postmortem hippocampal AD tissues. It was found that 12 h after LPS administration the expression of 231 genes in MH was significantly altered (FC > 2.0); however, after 96 h only the S100a8 gene encoding calgranulin A was activated (FC = 2.9). Gene ontology enrichment analysis demonstrated the alteration of gene expression related mostly to the immune-response including the gene *Lcn2* for Lipocalin 2 (FC = 237.8), involved in glia neurotoxicity. The expression of genes coding proteins involved in epigenetic regulation, histone deacetylases (*Hdac4*,*5*,*8*,*9*,*11*) and bromo- and extraterminal domain protein *Brd3* were downregulated; however, *Brd2* was found to be upregulated. Remarkably, the significant increase in expression of *Lcn2*, *S100a8*, *S100a9* and also *Saa3* and *Ch25h*, was found in AD brains suggesting that early changes of immune-response genes evoked by mild SIR could be crucial in AD pathogenesis.

## 1. Introduction

The endotoxin hypothesis of neurodegeneration assumes that systemic inflammatory response (SIR) evoked by bacterial endotoxin lipopolysaccharide (LPS) may trigger the cascade of signaling events in the brain leading to dysfunction, degeneration or death of neurons [1]. The immune system operates in close association with the nervous system [2]. Peripheral activation of immune system induces release of pro-inflammatory mediators that coordinate local and systemic response, but they also impact the central nervous system (CNS) leading to development of behavioral symptoms defined as “sickness behavior”. However, acute or chronic systemic inflammation may seriously affect the function of the CNS [3,4,5,6,7,8]. Moreover, the growing body of evidence indicates that SIR may contribute to pathogenesis/pathomechanism of Alzheimer’s disease (AD) and other neurodegenerative disorders [1,9,10,11,12,13,14]. It was recently suggested that LPS directly and indirectly stimulates microglia leading in consequence to synaptic and neuronal damage and phagocytosis [1]. In AD, LPS may accumulate in neocortical neurons and impair the efficient readout of neuronal genetic information necessary for the homeostasis and function of brain cells [15]. The endotoxin hypothesis of neurodegeneration proposes that decreasing LPS levels or LPS-induced neuroinflammation may protect the brain against neurodegenerative processes.

The level of soluble LPS in healthy human blood is very low (<1 EU/mL), but in some conditions, due to age, disease and increased intestinal epithelial permeability, it may significantly increase several-fold [16,17]. Similarly, several diseases are related to increased level of LPS in blood, like AD, atherosclerosis, amyotrophic lateral sclerosis, periodontitis, autism, diabetes, cardiovascular disease [1]. Moreover, aging-related changes of gut microbiota evoked increase of the level of endotoxin in blood plasma of mice [18]. A recent study by Palmer and co-workers demonstrated that naturally occurring subclinical endotoxemia leads to substantial changes in immune function in healthy individuals [19]. The main receptor for LPS is TLR4/MD2 complex, but also TREM2, RAGE, macrophage scavenger receptors, β2 integrins allow clearing of circulating LPS and trigger innate mechanisms of immune system leading to anti-bacterial response.

In experimental models, systemic administration of LPS is the most common method of activating innate mechanisms of immunity. Stimulation of TLR4 receptors on sensitive cells by LPS triggers the signaling cascade leading to activation of expression of several genes which are necessary for anti-microbial response. Our previous results demonstrated that hippocampus is highly sensitive and strongly affected by systemic administration of LPS. We demonstrated significant acute effects, including alterations of gene expression for inflammation–related proteins, activation of necrosis or cathepsin B-related autophagy, AIF-dependent apoptotic signaling, changes in ultrastructure of neuronal and glial cells [7,20]. Our recent study demonstrated also changes of calcium-dependent processes, like activation of calpains leading to dysregulation of cyclin-dependent kinase 5 (Cdk5) pathway [21]. Dysfunctional calcium signaling has been significantly implicated in neurodegenerative and neuroinflammatory processes in the CNS [22]. It was observed that even modest impairment of Ca^2+^ homeostasis may evoke significant functional alterations. The crucial role of altered Ca^2+^ homeostasis and signaling in the cognitive decline, disturbances in learning and memory during aging and the neurodegenerative disorders was previously indicated [22,23,24,25,26]. Glutamate receptors, voltage-operated calcium channels (VOCCs), sodium-calcium exchanger, acid-sensing ion channel-1, plasma membrane calcium ATPase, transient receptor potential (TRP) channels, all may contribute to the dysregulation of calcium homeostasis, leading to significant increase of cytosolic calcium concentration. High level of calcium activates several toxic or disadvantageous mechanisms, e.g., activation of nitric oxide synthase (NOS), lipases, proteases, kinases, endonucleases, leading finally to stress, energy crisis and disturbances in synaptic transmission. Calcium is also involved in regulating microglial transition from a “surveying” state to an “active” state [27]. The important role of calcium in regulating gene expression is well known [28], but recent studies demonstrated its involvement also in epigenetic mechanisms and splicing regulation [29,30,31,32]. The study by Yamawaki et al. suggested that epigenetic regulation of microglial function may play an important role during neuroinflammation in hippocampus [33]. For example, inhibitors of bromodomain and extra-terminal (BET) proteins, the readers of epigenetic code, have been shown to be effective inhibitors of microglia and macrophages [34,35].

Recent study by Hamasaki et al. demonstrated that sepsis enhanced expression of gene for S100a8 (calgranulin A) which is a protein involved in sensing Ca^2+^ signals and regulating Ca^2+^ homeostasis [36]. However, the transcriptional alterations of calcium-related proteins were never thoroughly investigated in the brain in conditions of systemic inflammation. Therefore, the aim of the present study was to evaluate the gene expression pattern in the mouse hippocampus 12 h and 4 days after single intraperitoneal administration of medium dose of LPS. Our microarray analysis revealed broad alterations in expression of several genes related to regulation of immune response and calcium homeostasis and signaling. These results were compared with parallel analysis of genes transcriptions in human post-mortem hippocampal AD tissue.

## 2. Results

Intraperitoneal administration of LPS induced in mice typical inflammatory response, body temperature alterations and sickness behavior, as reported previously [20]. Most of those changes were transient; they persisted only up to 48 h after LPS injection.

**Figure 1:**
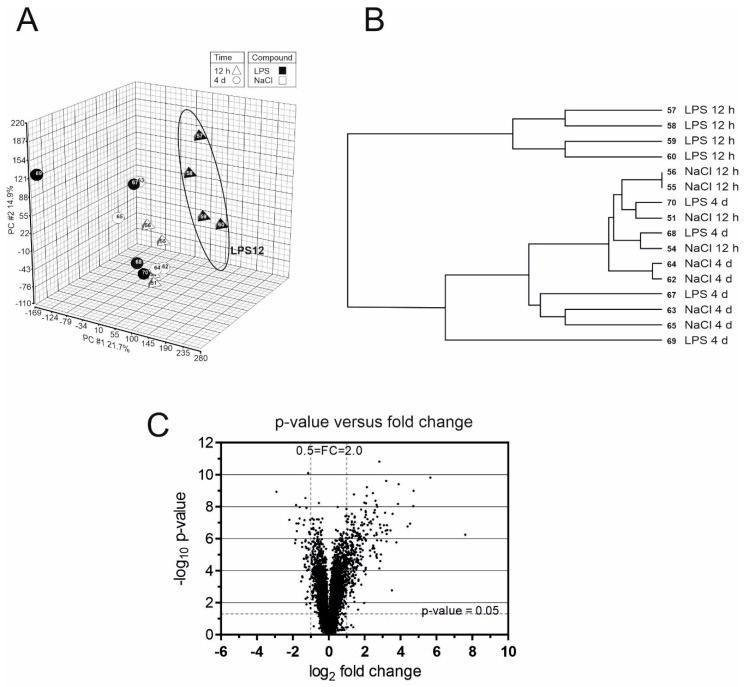
Analysis of global transcriptomic profiles. (**A**) Principal component analysis of gene expression profiles in hippocampi of mice treated with NaCl or lipopolysaccharide (LPS) for 12 h or 4 days. (**B**) The clustering analysis was based on the average linkage and Euclidean distances. (**C**) Volcano plot of the microarray data, plotting the negative log10 of the *p*-value against the log2 of the fold change. Twelve hours after administration of LPS the expression of 231 genes was significantly (*p* < 0.05, fold-change > 2.0) downregulated or upregulated, as compared to the control mice.

Microarray analysis of gene expression profile in hippocampus was performed in two time points—12 h and 4 days after intraperitoneal administration of LPS. Global transcriptomic profiles were analyzed by Principal Component Analysis (PCA) (Figure 1A) which demonstrated a clear separation between LPS-12 h group and other experimental groups. Also hierarchical clustering analysis of relative expression of genes in all samples demonstrated that LPS-12 h group significantly separated from other studied groups (Figure 1B). All up- and down-regulated genes were grouped into functional categories according to the Gene Ontology (GO) annotation by using Partek Genomics Suite 6.4 (Supplementary Figure S1). Gene ontology enrichment analysis showed that genes with altered expression level were most enriched in several groups related to immune response. The ten most numerous categories were: response to external stimulus (136 genes), response to wounding (120 genes), positive regulation of programmed cell death (119 genes), negative regulation of apoptosis (109 genes), inflammatory response (104 genes), response to abiotic stimulus (82 genes), negative regulation of cell proliferation (81 genes), GTPase activity (79 genes), regulation of cell activation (70 genes), regulation of leukocyte activation (70 genes).

The further analysis showed that 12 h after administration of LPS the expression of 231 genes was significantly changed (fold-change FC > 2.0) compared to the control mice (Figure 1C). Four days after administration of LPS expression of only one gene, *S100a8* (S100 calcium binding protein A8; calgranulin A), was significantly altered (*p* = 0.0465655, FC = 2.92642). The lists of ten the most increased and the most decreased transcripts at 12 h are presented in Table 1 (full list presented in Supplementary Table S1). It should be underlined that expression of gene coding Lipocain-2 is significantly upregulated by over 237-fold (Table 1). The full list of significantly (FDR < 0.05, FC > 2.0) up- or downregulated genes was subjected to core analysis by Ingenuity Pathway Analysis software (Ingenuity Systems) to identify the genes significantly associated with canonical pathways. In accordance to GO analysis, main pathways are related to the function of immune system (Supplementary Table S2).

**Figure 2:**
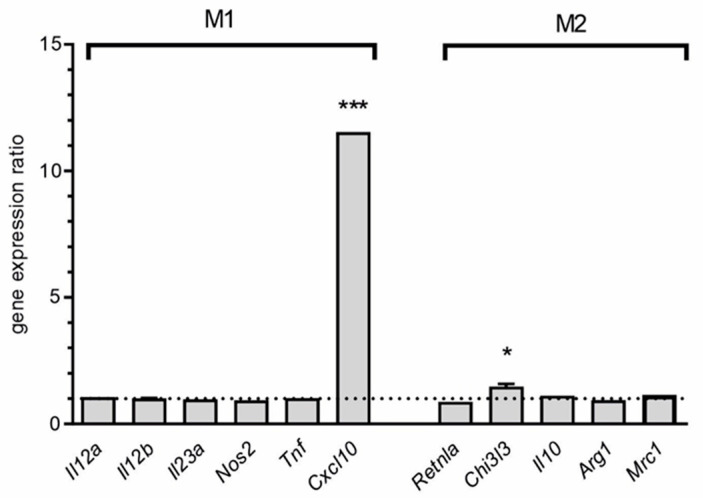
Microarray analysis of expression of neuroinflammation-related genes in hippocampus 12 h after peripheral administration of LPS. Markers of phenotype polarization. Mean (+SD) for all detected transcripts was presented. *n* = 4; * *p* < 0.05, ***p < 0.001, comparing to control group for at least one transcript.

In the brain, the immune system is represented mostly by microglial cells. To determine microglia phenotype polarization profile, the set of genes was analyzed [37]. As shown of Figure 2, among typical M1-related genes only *Cxcl10* was up-regulated. Interestingly, weak increase in mRNA level for M2-related *Chi3l3* gene was observed.

**Figure 3:**
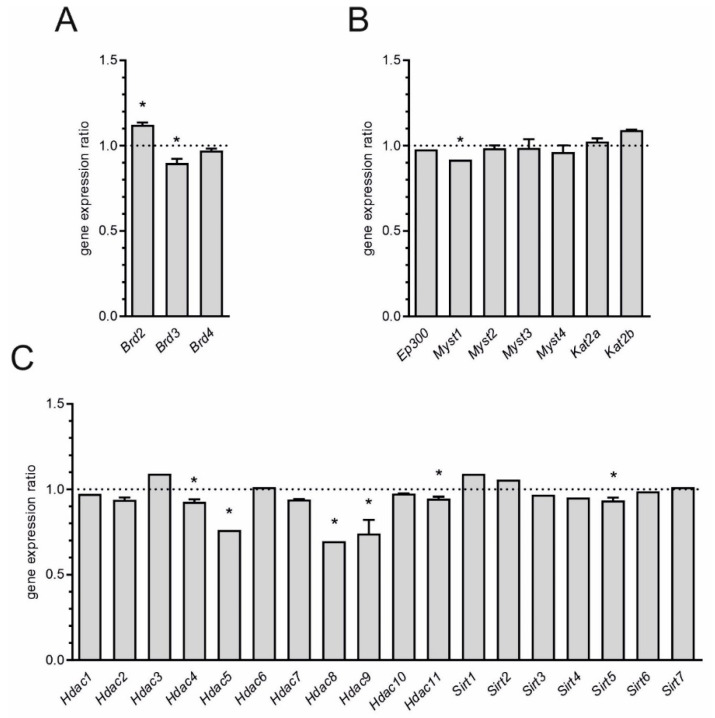
Microarray analysis of expression of genes for bromodomain and extra-terminal (BET) proteins and histone acetylation/deacetylation-related proteins in hippocampus 12 h after peripheral administration of LPS. (**A**) Bromodomain and extraterminal proteins (**B**) Histone acetylases, (**C**) Histone deacetylases. Mean (+SD) for all detected transcripts was presented. *n* = 4; * *p* < 0.05, comparing to control group for at least one transcript.

We analyzed also expression of genes related to epigenetic regulation, as emerging contributor to microglial plasticity (Figure 3) [38]. Among tested genes, we focused on those involved in histone acetylation code. We found that expression of bromodomain and extraterminal (BET) proteins, the readers of epigenetic code, was altered (Figure 3A). The level of mRNA for *Brd2* was increased, whereas expression of *Brd3* was decreased. Among analyzed histone acetylases, only the level of mRNA for *Myst1* was changed (Figure 3B). In the group of histone deacetylases (HDAC), expression of several genes was reduced: *Hdac4*, *Hdac5*, *Hdac8*, *Hdac9*, *Hdac11*, and *Sirt5* (Figure 3C).

**Figure 4:**
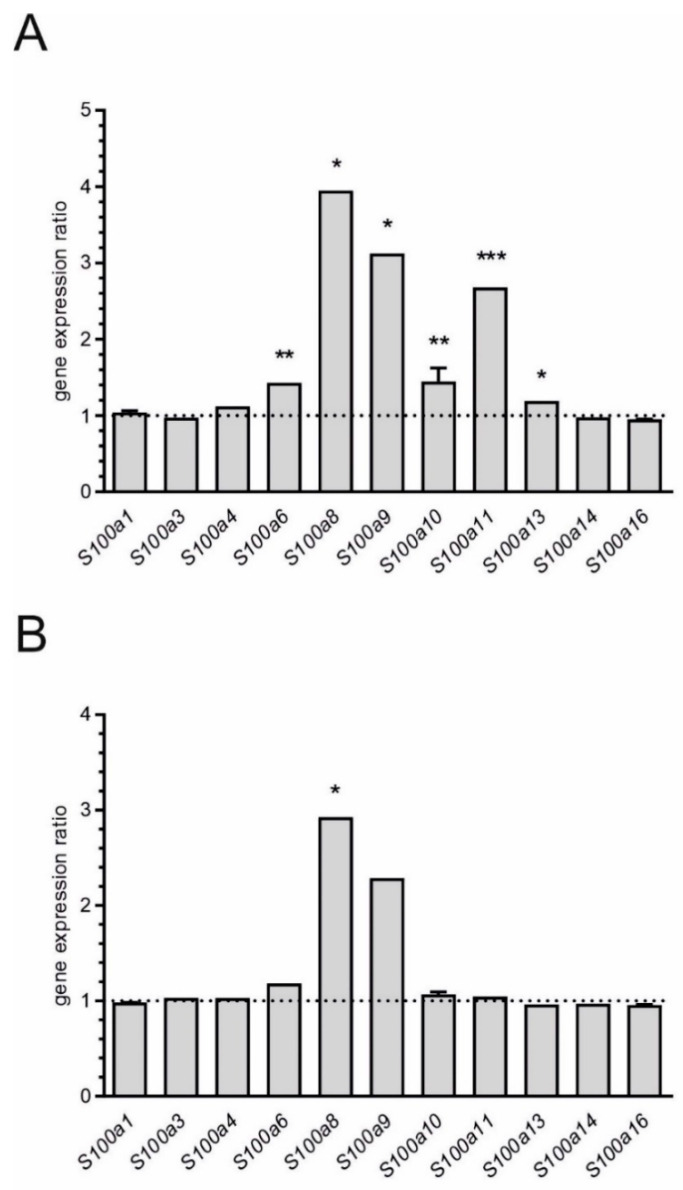
Microarray analysis of expression of genes for S100a family proteins in hippocampus 12 h (**A**) and 4 days (**B**) after peripheral administration of LPS. Mean (+SD) for all detected transcripts was presented. *n* = 4; * *p* < 0.05, ** *p* < 0.01, *** *p* < 0.001, comparing to control group for at least one transcript.

Then, we used the microarray data to identify changes in expression of genes for calcium-related proteins. We focused on S100a proteins. As shown of Figure 4, transcripts of 11 members of this family were detected by microarray analysis. Among them, statistically significant (*p* < 0.05) increase of expression of 6 genes was observed 12 h after administration of LPS. The highest increase was found for *S100a8* and *S100a9*. Four days after injection of LPS, expression of *S100a8* was still elevated but expression of *S100a9* was not changed (*p* = 0.115137).

**Figure 5:**
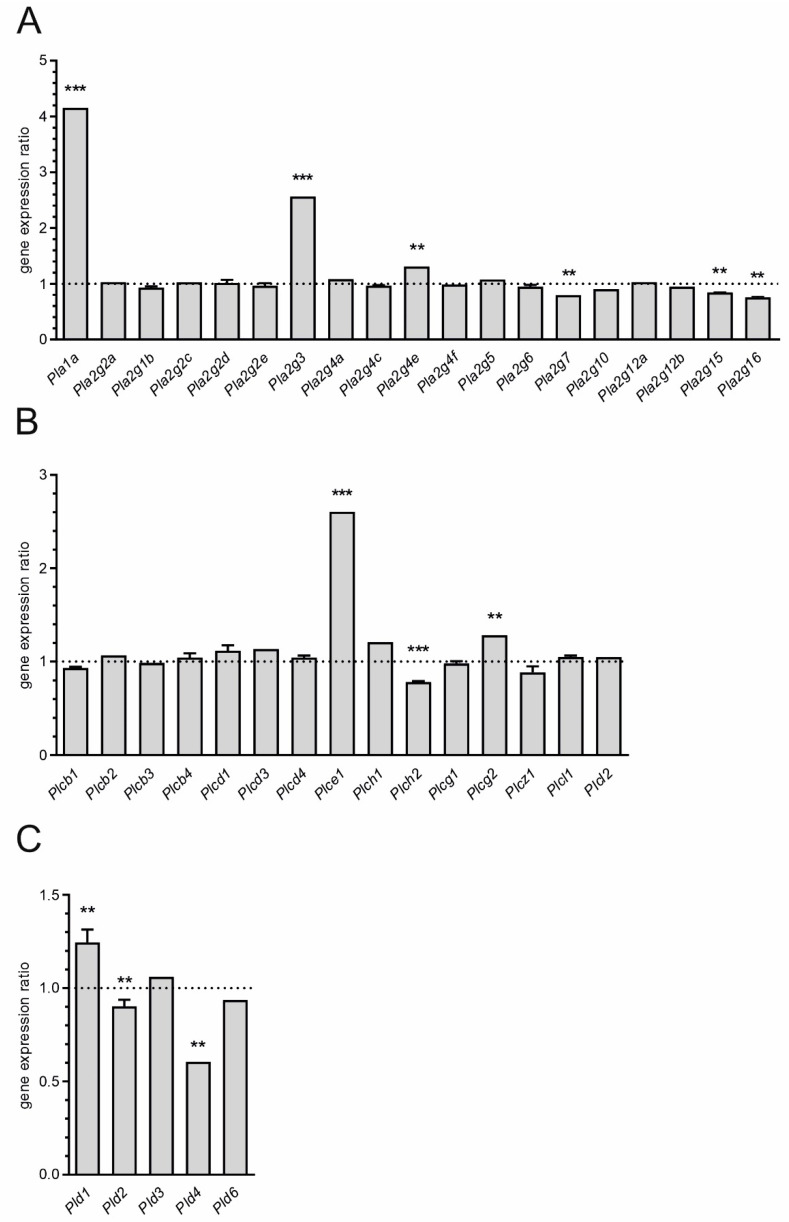
Microarray analysis of expression of genes for phospholipases in hippocampus 12 h after peripheral administration of LPS. (**A**) Expression of isoforms of phospholipase A2, (**B**) Expression of isoforms of phospholipase C, (**C**) Expression isoforms of phospholipase D. Mean (+SD) for all detected transcripts was presented. *n* = 4; ** *p* < 0.01, *** *p* < 0.001, comparing to control group for at least one transcript.

Finally, we analyzed expression of genes for phospholipases—the enzymes involved in membrane remodeling and signal transduction, many of which are calcium-dependent. Expression of 40 isoforms was detected (Figure 5). Among them, statistically significant increase of expression in hippocampus 12 h after administration of LPS was observed for *Pla1a*, *Pla2g3*, *Pla2g4e*, *Plce1*, *Plcg2*, and *Pld1*. Reduced expression was found for *Pla2g7*, *Pla2g15*, *Pla2g16*, *Plch2*, *Pld2*, and *Pld4*. 96 h after induction of SIR we did not observe significant changes.

**Figure 6:**
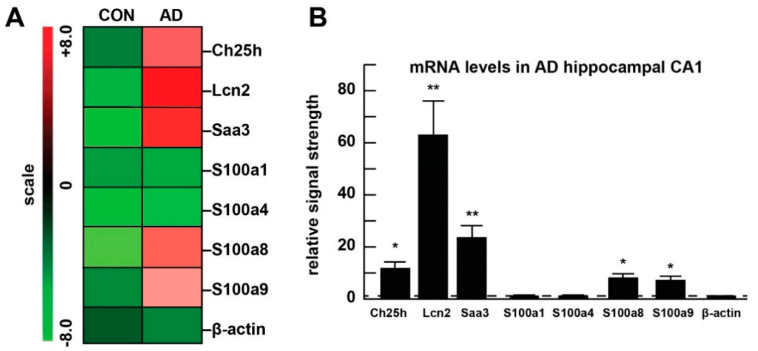
Microarray analysis of mRNA in Alzheimer’s disease (AD) brain. Hippocampal CA1 tissue samples from AD brains and age-matched controls (all female) were used for isolation of mRNA (Table 2). Analysis of mRNA was performed, as described above. The results of a mRNA-array based analysis of controls versus AD brains presented in cluster diagram “heat map” format (**A**) and in bar graph format (**B**). Compared to age-, gender- and post-mortem interval-matched controls, mRNA for *Ch25h*, *Lcn2*, *Saa3*, *S100a8*, and *S100a9* are significantly up-regulated in AD CA1 to levels 10- to 60-fold above controls. * *p* < 0.05, ** *p* < 0.01, comparing to control group.

Our parallel studies on human brain tissues using array-based mRNA analysis showed a significant up-regulation of *Ch25h*, *Lcn2*, *Saa3*, *S100a8*, and *S100a9* in AD hippocampus compared to age-, gender- and post-mortem interval (PMI)-matched controls (Figure 6).

## 3. Discussion

Our study indicated a relationship between SIR-evoked changes in readout of genetic information and AD-related gene expression. Twelve hours after systemic administration of LPS the expression pattern of 231 genes in mouse hippocampus was altered. However, 4 days after LPS only one gene coding S100 calcium binding protein A8 (*S100a8*) was significantly upregulated. In the present study, we provide evidence that 12 h after LPS injection, genes with altered expression were mostly related to the immune response. Among them, gene encoding Lipocalin 2 (*Lcn2*), considered as a neuro-inflammatogen, was most significantly activated (over 237.8 fold) in LPS-injected mice [39]. It was recently reported that Lcn2 regulates glial cell death/survival, morphology and motility [40,41]. Moreover, other research data demonstrated the significant role of Lcn2 as stimulator of chemokine release and neuronal cell death [42,43] in brain ischemia and several other pathological conditions [44,45,46].

The protective, antimicrobial action of Lcn2 is related to its binding to iron-loaded bacterial siderophores leading to their sequestration and, in consequence, iron-depletion as part of the acute-phase response to bacterial infection. It was proposed that Lcn2 promotes microglial M1 polarization [37]. It was observed in vitro that microglial expression and secretion of Lcn2 is increased after stimulation with LPS/IFN-γ (an M1-polarizing stimulus). In addition, expression of M1-related genes in microglia, but not M2-related genes, was significantly affected by overexpression or knockdown of *Lcn2* gene indicating essential role of this protein in regulation of microglial phenotype. Our data indicated sustained up-regulation of *Lcn2* expression 12 h post-injection, despite the expression of most M1-related genes which dropped to control level. However, one very important exception was observed in regard to an over 11-fold upregulation of transcription from the *Cxcl10* gene (Figure 2).

Recently, Lcn2 has emerged as an important component of pathophysiology of several disorders, including Alzheimer’s disease (AD), Parkinson’s disease, multiple sclerosis and depression [47,48,49,50]. It was suggested that Lcn2 may play some role in cognitive function during neuro-inflammation-related disorders [51]. As discovered in our microarray analysis of human post-mortem AD hippocampal tissue, the expression of the *Lcn2* gene is highly enhanced. As described above, Lcn2 is engaged in the neuro-inflammatory responses evoked by LPS and could lead to neurotoxic glial activation and/or functional polarization of glia cell [37,52]. Our analysis of gene markers of microglial phenotype polarization demonstrated higher expression (over 11-fold) of the gene encoding CXCL10 chemokine (M1 marker) and also about 1.5- fold upregulation of the gene *Chi3l3* (M2 marker). The data of Sui et al. [53] provide evidence that CXCL10 induces neuronal, caspase-dependent, apoptosis by Ca^2+^ ion dysregulation. Recent data by Bradburn et al. [54] showed that the CLXCL10 level is higher in older adults compared to young adults and is negatively associated with cognitive performance. This correlation was also found in AD where the protein level of this inflammatory cytokine was found to be significantly higher in prefrontal cortex of AD versus age-matched controls. It is known that chronic low–grade inflammation during aging may lead to disturbances of learning and memory. The hallmark of this inflammaging is an influx of leukocytes that is controlled by chemokine CXCL10 that play a key role in controlling the influx of the several leukocytes into the brain. This chemokine together with others exerted the effect through the binding and activation of CXCR3 receptor [55,56].

Four days after injection of LPS, expression of only one gene *S100a8* was significantly altered (FC = 2.93). The expression of another gene *S100a9* also had a tendency to increase, however the result was not statistically significant. S100a8 is calcium-binding protein belonging to the S100 family. Although S100 family proteins literally are not cytokines, they have similar functions, stimulating leukocyte recruitment and inducing cytokine secretion. Moreover, S100 proteins appear to regulate learning and memory function. Recent studies have demonstrated the significant role of seven proteins from S100 family, including S100a8 and S100a9, in the pathomechanism of AD [57]. Our data demonstrated significant enhancement of gene expression of *S100a6*, *S100a8*, *S100a9*, *S100a10*, *S100a11* and *S100a13* 12 h after injection of LPS. The previous data mentioned that S100a8 and S100a9 induced inflammation signaling processes by activation of TLR4 receptor and MAPKs [58]. Both of these proteins, via a signaling pathway involving TLR4, RAGE and ERK/NF-κB, lead to secretion of TNF-α and IL-6 in microglia cells (BV2) [59]. It was also indicated that S100a8/S100a9, through this signaling pathway, may induce the transcriptional activity of β-secretases 1 and 2 (BACE1 and BACE2) and could be involved in amyloid β (Aβ) peptide generation [60]. Our results further indicated that expression of S100a8 and S100a9 is increased in postmortem human tissue samples from AD hippocampus (Figure 6). Other data has indicated that aggregation of S100a8 precedes Aβ plaque formation in AD transgenic (Tg) mice [61]. Also S100a9 induces Aβ fibrilization [62]. As summarized by Wang et al. [58], these changes can be an important link between the Aβ peptide cascade and neuroinflammatory processes.

Our data further demonstrated broad changes in expression of genes encoding proteins involved in epigenetic regulation of transcription, mainly histone deacetylases and BET proteins. The significant role of increased acetylation of histone H3 in regulating transcription of pro-inflammatory genes and, in consequence, in neuronal and microglial neuro-inflammatory response, was demonstrated in the rat hypothalamus and hippocampus after peripheral administration of LPS [63]. However, inhibitors of HDAC were shown to suppress expression of pro-inflammatory mediators in glial cultures exposed to LPS [64]. Also, alterations of expression of genes for BET proteins could contribute to the activation of microglia. Recent studies have highlighted the importance of genetic/epigenetic phenomena and suggested that they contribute more to AD than previously expected [65,66]. Acetylation-deacetylation of DNA-bound proteins is a crucial epigenetic mechanism controlling the structure of chromatin and genetic activity. Bromodomain and extra-terminal domain (BET) proteins are “readers” of protein acetylation. While two highly conserved N-terminal bromodomain (BD) modules are involved in recognizing acetylated histone tails and other acetylated proteins, the extra-terminal domain (ET) has been implicated in protein-protein interaction. Therefore, nuclear BET proteins recognize acetylated lysine groups in histones and initiate formation of multi-protein complexes involved in controlling activation or suppression of the expression of numerous genes involved in the cell cycle, growth, inflammation, and cancer [67,68]. In humans (similar to the mouse proteome) there are four proteins of this family: Brd2, Brd3, Brd4 and BrdT, but expression of the latter isoform is limited to the male germ line [69]. Several studies demonstrated the interaction of BET proteins with transcription factors like NF-κB, E2F-1, AP-1 etc., [35,70,71,72,73,74,75] and other DNA-bound proteins, like poly (ADP-ribose) polymerase-1 (PARP-1) [76]. A growing body of evidence indicates that epigenetic control mechanisms are involved in regulating the immune system. Recent studies confirmed that BET proteins may affect the progression of inflammation and demonstrated that they are critical for macrophage activation [35,77,78,79,80]. Brd2 and Brd4 physically associate with the promoters of inflammatory cytokine genes, reducing production of cytokines TNFα, IL-6, MCP-1 in vitro and in vivo. Also, one study on the transgenic model of AD (3×Tg mice) confirmed the important function of BET proteins in regulating brain gene expression in neuro-inflammation-related conditions, including AD [80]. Importantly, *Brd3* gene is Ca^2+^-responsive gene [81].

Systemic inflammatory response (SIR) evoked by the bacterial endotoxin, LPS, induces significant alterations in gene transcription profiles in the hippocampus, the part of brain which is the most important for learning and memory processes. This LPS-evoked inflammation leads to gigantic enhancement of expression of gene coding Lcn2 that could be responsible for microglia polarization and toxicity. Then upregulation of the gene coding chemokine CXCL10 that is typical for M1 microglia may lead to influx of leukocytes, elevation of Ca^2+^, activation of CXCR3 receptor and Ca^2+^/caspase-dependent cells death. This chemokine can be also expressed by neurons and stromal cells. It is widely accepted that Ca^2+^ dysregulation plays a significant role in alterations of learning and memory in the brain during aging, AD and other pathological conditions. Calcium signaling is involved in most physiological processes and even negligible alterations of Ca^2+^ homeostasis may evoke significant functional changes. Our data indicated that SIR affects transcription of several phospholipases, among them four are calcium-dependent: the phospholipase A2 group III (PLA2G3) and group IVE (PLA2G4E), the phospholipase C isoform epsilon-1 (PLCE) and eta-2 (PLCL4). Phospholipases are a family of lipolytic enzymes that regulate composition of cellular membranes, but also play important role in controlling cellular activities [82]. Liberated by phospholipases, arachidonic acid (AA) and its metabolites are significantly engaged in mechanism of learning and memory [83,84]; therefore, substantial changes in their expression may also significantly impact the brain’s function. Our previous studies indicated several morphological and molecular changes, including those at the mRNA level, for PLA2 in hippocampus after SIR evoked by LPS [7]. Additionally, we have also previously reported the impairment of cognitive function after LPS-evoked SIR, but the molecular basis is not fully understood [85,86].

It is not fully clear how peripheral LPS induces molecular processes within the brain. In common opinion, LPS affects the brain in complex mechanisms linking direct and indirect effects. There are several possible pathways. One option is that LPS directly or by inducing systemic inflammation affects function of the blood brain barrier (BBB) enabling diffusion of LPS from blood plasma directly to brain parenchyma [87]. The data indicate that LPS induces a concentration- and time-dependent opening of the BBB. Our previous study on the same experimental model demonstrated that 48 h after i.p. administration of LPS at 1 mg/kg degeneration of microcapillaries occurred [6]. We observed pronounced ultrastructural changes in capillary vessels: constriction of capillary lumen by swollen endothelial cell without changes in capillary basement membrane. However, LPS which in blood is usually bound to lipoproteins may be also transported through BBB via lipoprotein transport mechanisms [88]. It is impossible to exclude that some LPS molecules could translocate into the brain region where BBB does not exist (as circumventricular organs, the roof of third and fourth ventricles, the roof of diencephalon etc.). Therefore, LPS may reach brain parenchyma even if BBB in the major part of the brain is intact. Other possibility is that LPS directly, by activating specific receptors on BBB, or indirectly, by peripheral mediators of inflammation, activates cells of BBB which in turn release cytokines into the brain parenchyma [87,89]. Finally, LPS may activate peripheral nerves or induce peripheral cytokine storm that affects the brain.

Our microarray analysis of human post-mortem hippocampal tissue demonstrated that expression of several genes which were activated in brains of LPS-injected mice were also increased in postmortem AD tissue samples from the hippocampus. The significant increase in the *Lcn2* is noteworthy as Lcn2 has been recently implicated in mediating neuronal damage in vascular brain injuries and AD and other progressive neurodegenerative dementias, and may be involved in facilitating the progression of AD-type change [46,90]. The genes *Saa3* and *Ch25h*, whose expression was significantly increased in AD brains, were significantly activated during systemic inflammation in hippocampus of mouse. Both genes are involved in lipid metabolism, suggesting that inflammation-related lipid disturbances may contribute to AD pathology and may be a strategic target for potential therapies [91].

**Figure 7:**
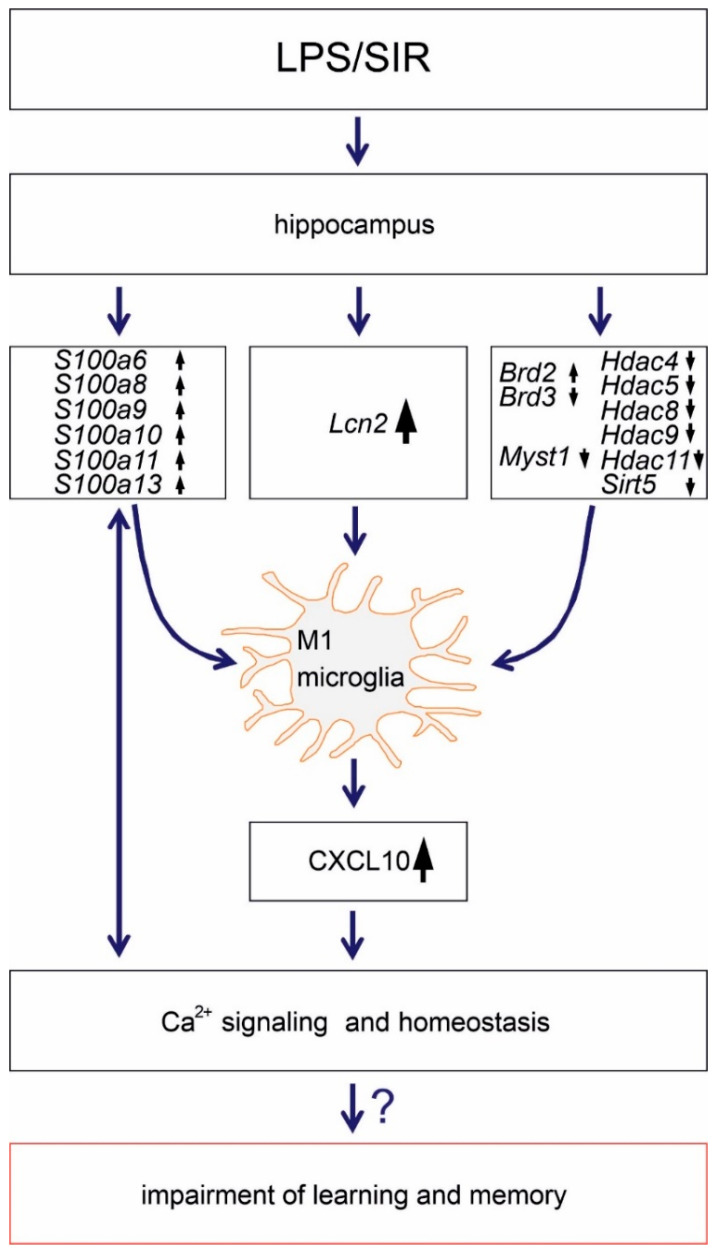
Schematic representation of main findings of the study. Arrows 
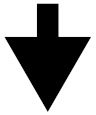
 and 
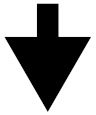
 indicate an increase and a decrease in gene expression, respectively.

Although previous studies have demonstrated the impact of systemic inflammation on gene expression in the brain, our current data provide insight into the global gene expression pattern in hippocampus after acute and mild SIR. These results are important for understanding hippocampal sensitivity to stress and may indicate novel therapeutic targets for age- and neuro-inflammation-related disorders (Figure 7).

## 4. Materials and Methods

### 4.1. Animals

All experiments were carried out on male, 2–3-month-old (20–25 g), C57BL/6 mice, supplied by the Animal House of Mossakowski Medical Research Centre Polish Academy of Sciences (Warsaw, Poland). The animals were maintained under controlled temperature (22 °C ± 10%) and humidity (55% ± 10%) conditions on a 12-h light/dark cycle. All of the experiments conducted on the animals were approved by the IV Local Ethics Committee for Animal Experimentation in Warsaw (permission 26/2007; 18 July 2007), were carried out in accordance with the EU Directive 2010/63/EU for animal experiments, and comply the ARRIVE guidelines. All efforts were made to minimize animal suffering and to reduce the number of animals used. All manipulations were performed gently and quickly to reduce animal’s stress. LPS (from *E. coli* serotype O55:B5; toxicity 1.5×10^7^ EU/mg; Sigma, St. Louis, USA) was dissolved in saline and administered intraperitoneally (i.p.) in a dose of 1 mg/kg, an appropriate volume of the solvent (100 µL) was injected (i.p.) to control animals. Following this, 12 h and 4 days after administration of LPS, animals were deeply anaesthetized with sodium pentobarbital (60 mg/kg b.w., i.p. Polfa, Pulawy, Poland) and perfused transcardially with 0.1 M sodium phosphate buffer, pH 7.4 at 4 °C to remove blood from the brain. Then, brains were collected and analyzed.

### 4.2. Ethical Compliance

The acquisition, handling, experimental, and analytical procedures involving postmortem human brain tissues were carried out in an ethical manner in strict accordance with the ethics review board policies at brain and tissue donor institutions and at the Louisiana State University (LSU) Health Sciences Center. Informed consent from next of kin was obtained at brain and tissue donor institutions for all tissue samples prior to autopsy and donation; coded postmortem brain tissue samples (containing no personal identifying information of the donors) were obtained from the brain and tissue banks listed above. The ethical use of postmortem human brain tissues and their analyses were also carried out in strict accordance with the Institutional Biosafety Committee and the Institutional Review Board Committee (IBC/IRBC) ethical guidelines IBC#18059 and IRBC#6774 at the LSU Health Sciences Center, New Orleans LA 70112 USA.

### 4.3. Microarray Analysis of Murine Brain RNA and Statistical Analysis

Total RNA was isolated from the mouse hippocampus using the RNeasy Lipid Tissue Mini Kit (Qiagen, Hilden, Germany). Microarray analysis was performed in the Laboratory of Microarray Analysis of Center of Excellence BioExploratorium, Department of Biology Warsaw University and Institute of Biochemistry and Biophysics PAS, Warsaw, Poland, using a standard protocol provided by Affymetrix. RNA quality was established using the Agilent 2100 Bioanalyzer and RNA 6000 Nano Chip Kit (Agilent Technologie, Santa Clara, CA, USA). For expression analysis the Affymetrix Gene Chip Mouse genome 430 2.0 were used. Synthesis of cDNA was carried out starting from 2 μg of total RNA (One-Cycle cDNA Synthesis Kit, Affymetrix Inc., Santa Clara, CA, USA). For synthesis of biotinylated cRNA (IVT Labeling Kit, Affymetrix) 12 μL of purified (GeneChip Sample Cleanup Module) double-stranded cDNA were used. Labelled cRNA was purified (GeneChip Sample Cleanup Module, Qiagen, Valencia, CA, USA), fragmented and hybridized with genome array. Washing, staining with streptavidin-phycoerythrin conjugate and scanning of the arrays in Affymetrix GeneChip 3000 scanner were performed according to recommendation of Affymetrix Gene Expression Analysis Technical Manual. Quality controls were performed according to the manufacturer’s recommendations.

In the mouse gene expression studies microarray data acquired by Affymetrix GeneChip Scanner 3000 was converted to CEL files with use of Affymetrix Expression Console Software. CEL files were imported to Partek Genomics Suite 6.4 (Partek Inc., St. Louis, MO, USA) for statistical analysis. Data were normalized with GC-RMA method and log2 transformed. A three-way ANOVA was performed and genes with an FDR < 0.05 and FC > 2 were considered significantly altered in their expression. Probes lacking gene names, designated as cDNA and those described as hypothetical were removed from the lists. Lists of genes showing significant differences in expression levels between groups were submitted to Ingenuity Pathway Analysis (Ingenuity^®^ Systems, www.ingenuity.com) for canonical pathways analysis and subjected to network analyses.

### 4.4. Microarray Analysis of Human Brain RNA and Statistical Analysis

Analysis of Human hippocampal CA1-AD and age- and gender-matched control human hippocampal CA1 tissues were obtained from brain and tissue repositories including the LSU Health Sciences Center archives, New Orleans LA, USA; the Harvard Brain Tissue Bank, Boston MA, USA; the University of Oregon Health Sciences Center (OHSC), Portland OR, USA; the National Disease Research Interchange (NDRI), Philadelphia PA, USA; National Institute of Health (NIH) Bethesda MD, USA collaborators and researchers, and by the Institute for Memory Impairments and Neurological Disorders and the University of California at Irvine Alzheimer’s Disease Research Center (MIND-UCI-ADRC) Irvine CA, USA; funding for the UCI-ADRC was provided by NIH/NIA Grant P50 AG16573.

Gene expression analysis of human post-mortem brain samples by DNA arrays—A guanidine isothiocyanate- and silica gel-based membrane total RNA purification system and miRNA isolation kit (PureLink™ Invitrogen, Carlsbad, CA) were used to isolate total RNA for DNA array-based analysis; total RNA concentrations were quantified using RNA 6000 Nano LabChips and a 2100 Bioanalyzer (Caliper Technologies, Mountainview, CA; Agilent Technologies, Palo Alto, CA). Ch25h, Lcn2, Saa3, S100a and β-actin cytoskeletal RNA abundances were analyzed and quantified using GeneCHip arrays (LC Sciences, Houston TX, USA) or Northern dot blot arrays as previously described [92,93,94,95,96]. Altered RNA levels of interest were further verified using a quantitative Northern dot blot focusing assay that utilizes a T4 PNK kinase radiolabel system employing [α-32P]-dATP (6000 Ci/m mol; Invitrogen, Carlsbad, CA) that significantly interrogates the abundance of RNA and miRNA signals [94,96].

In the human gene expression studies, relative DNA array signal strengths were quantified using data-acquisition software provided with a GS250 molecular imager (Bio-Rad, Hercules, CA) or proprietary bioinformatics software (LC Sciences) and graphic presentations (including heat maps and comparative bar graphs) were performed using Excel algorithms (Microsoft, Seattle, WA) and Adobe Photoshop 6.0 (Adobe Systems, San Jose CA). Statistical significance was analyzed using a two-way factorial analysis of variance (*p*, ANOVA; SAS Institute, Cary, NC). A “*p*” value of <0.05 was deemed as statistically significant; experimental values in the figures are expressed as means +/− one standard deviation (SD) of that mean.

### 4.5. Experimental Design

To avoid bias, allocation to experimental groups was randomized, and samples were analyzed in random order. Investigators were unaware of assigned sample designations until completion of the experiment.

## Figures and Tables

**Table 1:** Summary of genes with highest up- or downregulation 12 h after systemic administration of LPS.

Part A: List of 10 the Most Upregulated Genes (FDR < 0.05)		
**Gene Symbol**	**Gene Title**	**FC**
*Lcn2*	lipocalin 2	237.80
*Saa3*	serum amyloid A 3	53.09
*Ms4a6d*	membrane-spanning 4-domains, subfamily A, member 6D	26.23
*Gbp2*	guanylate binding protein 2	22.32
*Ctla2a*	cytotoxic T lymphocyte-associated protein 2 alpha	15.50
*Ccl5*	chemokine (C-C motif) ligand 5	14.31
*Cxcl10*	chemokine (C-X-C motif) ligand 10	12.15
*Lrg1*	leucine-rich alpha-2-glycoprotein 1	11.34
*Ch25h*	cholesterol 25-hydroxylase	10.43
*Ctla2b*	cytotoxic T lymphocyte-associated protein 2 beta	10.01
**Part B: List of 10 the Most Downregulated Genes (FDR < 0.05)**		
**Gene Symbol**	**Gene Title**	**FC**
*Gpr34*	G protein-coupled receptor 34	−7.85
*P2ry12*	purinergic receptor P2Y, G-protein coupled 12	−4.61
*Serpinb1a*	serine (or cysteine) peptidase inhibitor, clade B, member 1a	−3.75
*Akr1c14*	aldo-keto reductase family 1, member C14	−3.56
*Itm2a*	integral membrane protein 2A	−3.51
*Cxcl12*	chemokine (C-X-C motif) ligand 12	−3.45
*Ugt8a*	UDP galactosyltransferase 8A	−3.10
*Tek*	endothelial-specific receptor tyrosine kinase	−3.06
*Slco1c1*	solute carrier organic anion transporter family, member 1c1	−2.88
*Slc40a1*	solute carrier family 40 (iron-regulated transporter), member 1	−2.71

FDR- False Discovery Rate.

**Table 2:** Summary of hippocampal CA1 tissues from control and Alzheimer’s groups used in this study.

Group	*n*	Age x+/−SD	Age Range	Mean PMI *^1^	RNA A_260/280_	RNA 28S/18S	RNA Yield *^2^
Control (CON)	30	71.5+/−6.1	64–77	3.0	2.10	1.50	1.3
Alzheimer (AD)	36	72.2+/−7.6	66–79	3.1	2.09	1.45	1.3

*n* = number of individual brain samples; to further understand rends in gene expression the current gene expression analysis was based on a pool of total RNA isolated from *n* = 30 controls (CON) and *n* = 36 Alzheimer’s disease (AD) cases; age is time of death in years; age range indicates range of the individual means; post-mortem interval (PMI; death to brain freezing interval) range is the range in mean in hours. RNA_A260/280_ and RNA 18S/28S mean ratios are indicative of high brain tissue RNA spectral quality [97,98,99]. There was no significant difference between the mean yield of total RNA between the control or AD tissues. Characterization of control and AD total RNA message; *^1^ mean death to brain freezing interval in hours at −81 °C; *^2^ average yield in total µg RNA/mg wet weight brain tissue.

## Supplementary Materials

The following are available online at https://www.mdpi.com/1422-0067/21/21/7838/s1, Figure S1: Functional categories represented by genes up- or downregulated by LPS (12 h), Table S1: The list of all genes up- or downregulated 12 h after systemic administration of LPS (FDR < 0.05; FC > 2), Table S2: List of the genes in most significantly altered top ten canonical pathways.
