# Supplementary material for: Acute Systemic Inflammatory Response Alters Transcription Profile of Genes Related to Immune Response and Ca2+ Homeostasis in Hippocampus; Relevance to Neurodegenerative Disorders
Source: Int J Mol Sci. 2020 Oct 22;21(21):7838. doi: 10.3390/ijms21217838 (PMC7660108; doi:10.3390/ijms21217838)
Supplement: Supplementary file 1 [file ijms-21-07838-s001.pdf]

Supplementary materials

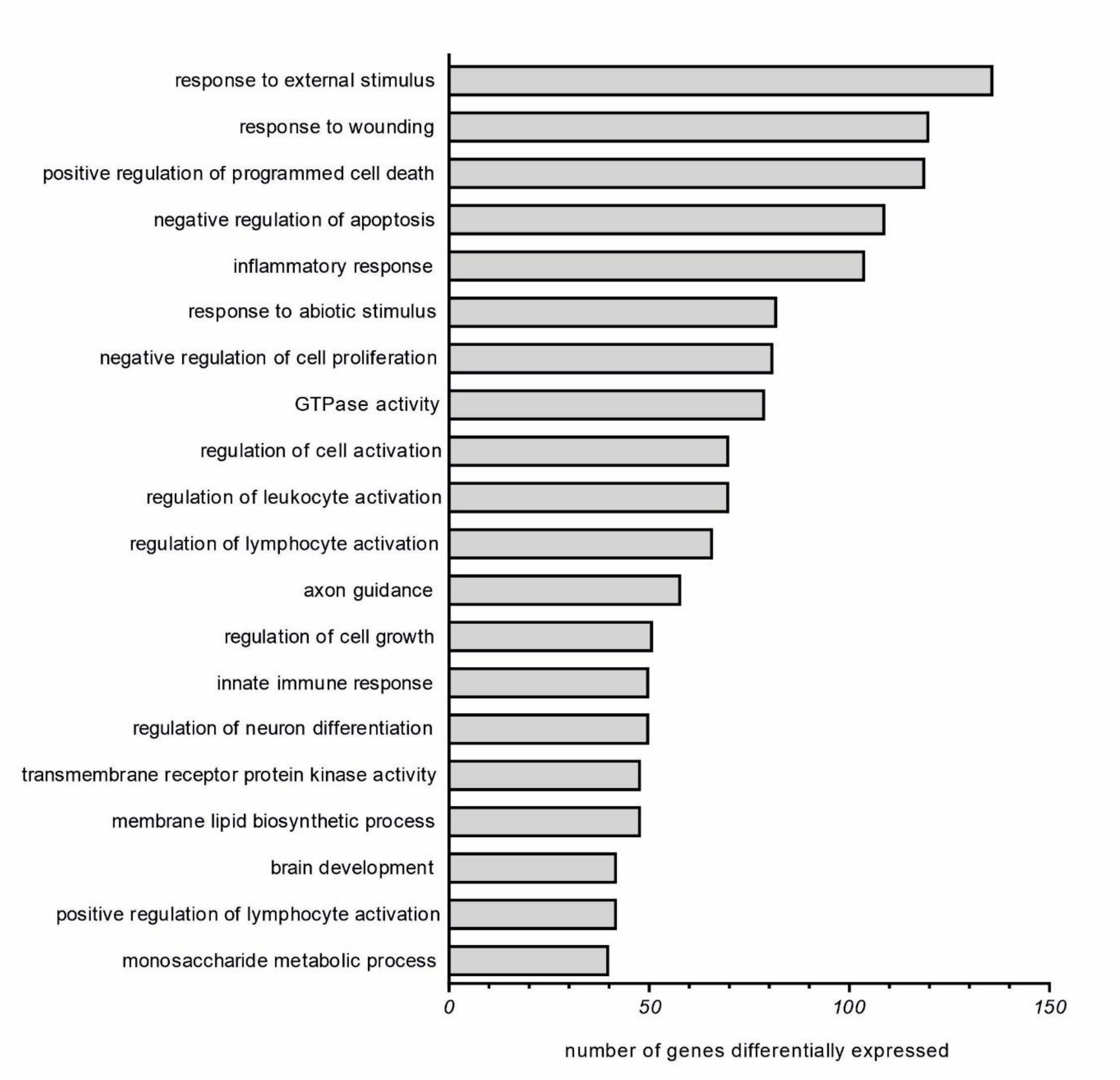

Supplementary Figure 1. Functional categories represented by genes up- or downregulated by LPS (12 h). Top 20 functional categories with p values <0.01 are shown.

Supplementary Table 1. The list of all genes up- or downregulated 12 h after systemic administration of LPS (FDR<0.05; FC>2)

Part A: List of all upregulated genes

**Gene Symbol**	**Gene Title**	FC
Lcn2	lipocalin 2	237.80
Saa3	serum amyloid A 3	53.09
Ms4a6d	membrane-spanning 4-domains, subfamily A, member 6D	26.23
Gbp2	guanylate binding protein 2	22.32
Ctla2a	cytotoxic T lymphocyte-associated protein 2 alpha	15.50
Ccl5	chemokine (C-C motif) ligand 5	14.31
Cxcl10	chemokine (C-X-C motif) ligand 10	12.15
Lrg1	leucine-rich alpha-2-glycoprotein 1	11.34
Ch25h	cholesterol 25-hydroxylase	10.43
Ctla2b	cytotoxic T lymphocyte-associated protein 2 beta	10.01
Mpa2l	macrophage activation 2 like	9.27
S3-12	plasma membrane associated protein, S3-12	9.19
Timp1	tissue inhibitor of metalloproteinase 1	8.93
Ms4a6b	membrane-spanning 4-domains, subfamily A, member 6B	8.91
A2m	alpha-2-macroglobulin	8.80
Osmr	oncostatin M receptor	7.58
Ifitm3	interferon induced transmembrane protein 3	7.15
Mmp3	matrix metallopeptidase 3	7.03
Iigp1	interferon inducible GTPase 1	6.98
Prg4	proteoglycan 4	6.42
Spp1	secreted phosphoprotein 1	6.42
Igtp	interferon gamma induced GTPase	6.41
Rhoj	ras homolog gene family, member J	6.10
Oasl2	2'-5' oligoadenylate synthetase-like 2	5.70
Ms4a6c	membrane-spanning 4-domains, subfamily A, member 6C	5.57
Saa1	serum amyloid A 1	5.45
Gbp3	guanylate binding protein 3	5.44
Socs3	suppressor of cytokine signaling 3	5.32
Steap4	STEAP family member 4	5.24
Apod	apolipoprotein D	5.20
Cdkn1a	cyclin-dependent kinase inhibitor 1A (P21)	5.18
Serpina3n	serine (or cysteine) peptidase inhibitor, clade A, member 3N	5.10
Ifitm1	interferon induced transmembrane protein 1	5.01
Cp	ceruloplasmin	4.81
Xdh	xanthine dehydrogenase	4.72
Pglyrp1	peptidoglycan recognition protein 1	4.64
Tubb6	tubulin, beta 6	4.39

Icam1	intercellular adhesion molecule 1	4.36
Srgn	serglycin	4.35
Slc10a6	solute carrier family 10 (sodium/bile acid cotransporter family), member 6	4.32
Angptl4	angiopoietin-like 4	4.23
Ccl12	chemokine (C-C motif) ligand 12	4.22
Pla1a	phospholipase A1 member A	4.21
Galntl2	UDP-N-acetyl-alpha-D-galactosamine:polypeptide N-acetylgalactosaminyltransferase	4.16
Ly6a	lymphocyte antigen 6 complex, locus A	4.14
Map3k6	mitogen-activated protein kinase kinase kinase 6	4.09
Mt2	metallothionein 2	4.01
Ifitm2	interferon induced transmembrane protein 2	3.94
Cxcl1	chemokine (C-X-C motif) ligand 1	3.91
Ucp2	uncoupling protein 2 (mitochondrial, proton carrier)	3.86
Igsf6	immunoglobulin superfamily, member 6	3.77
Ada	adenosine deaminase	3.77
Tgm2	transglutaminase 2, C polypeptide	3.65
Hspb1	heat shock protein 1	3.61
Irf7	interferon regulatory factor 7	3.56
Gpr65	G-protein coupled receptor 65	3.53
Ifit3	interferon-induced protein with tetratricopeptide repeats 3	3.50
Fcgr2b	Fc receptor, IgG, low affinity IIb	3.47
Adamts9	a disintegrin-like and metallopeptidase (reprolysin type) with thrombospondin type 1 motif, 9	3.45
Hspb6	heat shock protein, alpha-crystallin-related, B6	3.41
Gbp6	guanylate binding protein 6	3.39
Msr1	macrophage scavenger receptor 1	3.36
Rtp4	receptor transporter protein 4	3.36
Fcgr4	Fc receptor, IgG, low affinity IV	3.35
Irgm1	immunity-related GTPase family M member 1	3.35
Pmaip1	phorbol-12-myristate-13-acetate-induced protein 1	3.32
Rsad2	radical S-adenosyl methionine domain containing 2	3.27
Cyp1b1	cytochrome P450, family 1, subfamily b, polypeptide 1	3.23
Cd44	CD44 antigen	3.22
Adamts1	a disintegrin-like and metallopeptidase (reprolysin type) with thrombospondin type 1 motif, 1	3.22
Arrdc2	arrestin domain containing 2	3.22
Ifit1	interferon-induced protein with tetratricopeptide repeats 1	3.19
Sult1a1	sulfotransferase family 1A, phenol-preferring, member 1	3.19
Maff	v-maf musculoaponeurotic fibrosarcoma oncogene family, protein F (avian)	3.18
Glipr2	GLI pathogenesis-related 2	3.12

Trim30	tripartite motif-containing 30	3.07
Ifi44	interferon-induced protein 44	3.06
Acer2	alkaline ceramidase 2	3.05
Pp11r	placental protein 11 related	3.03
Iigp2	interferon inducible GTPase 2	3.02
Ifi47	interferon gamma inducible protein 47	3.01
Cd14	CD14 antigen	3.00
Psmb8	proteasome (prosome, macropain) subunit, beta type 8 (large multifunctional peptidase 7)	2.96
Serping1	serine (or cysteine) peptidase inhibitor, clade G, member 1	2.96
Sgk3	serum/glucocorticoid regulated kinase 3	2.95
Gpr84	G protein-coupled receptor 84	2.95
Lgals3bp	lectin, galactoside-binding, soluble, 3 binding protein	2.94
Plaur	plasminogen activator, urokinase receptor	2.92
Nfkbia	nuclear factor of kappa light polypeptide gene enhancer in B-cells inhibitor, alpha	2.91
Cd93	CD93 antigen	2.90
Sgk1	serum/glucocorticoid regulated kinase 1	2.89
Tap1	transporter 1, ATP-binding cassette, sub-family B (MDR/TAP)	2.86
Ccl9	chemokine (C-C motif) ligand 9	2.83
Tspan4	tetraspanin 4	2.79
Scara5	scavenger receptor class A, member 5 (putative)	2.78
Slfn4	schlafen 4	2.77
Apold1	apolipoprotein L domain containing 1	2.76
Agxt2l1	alanine-glyoxylate aminotransferase 2-like 1	2.76
Crispld2	cysteine-rich secretory protein LCCL domain containing 2	2.75
Fcgr3	Fc receptor, IgG, low affinity III	2.75
C5ar1	complement component 5a receptor 1	2.74
Igfbp7	insulin-like growth factor binding protein 7	2.70
Bcl2a1a	B-cell leukemia/lymphoma 2 related protein A1a	2.70
Aspg	asparaginase homolog (S. cerevisiae)	2.66
Csf3	colony stimulating factor 3 (granulocyte)	2.64
Sdc4	syndecan 4	2.62
Ctsc	cathepsin C	2.62
S100a11	S100 calcium binding protein A11 (calgizzarin)	2.62
Xaf1	XIAP associated factor 1	2.62
Scgb3a1	secretoglobin, family 3A, member 1	2.61
Fkbp5	FK506 binding protein 5	2.61
Pla2g3	phospholipase A2, group III	2.59
Ier3	immediate early response 3	2.59
Ggta1	glycoprotein galactosyltransferase alpha 1, 3	2.58

Il2rg	interleukin 2 receptor, gamma chain	2.57
Gimap6	GTPase, IMAP family member 6	2.57
Ptx3	pentraxin related gene	2.57
Tlr2	toll-like receptor 2	2.56
Gp49a	glycoprotein 49 A	2.56
Emp1	epithelial membrane protein 1	2.56
C3	complement component 3	2.56
Herc5	hect domain and RLD 5	2.56
Cebpd	CCAAT/enhancer binding protein (C/EBP), delta	2.55
Kcna5	potassium voltage-gated channel, shaker-related subfamily, member 5	2.55
Cd86	CD86 antigen	2.54
Txnip	thioredoxin interacting protein	2.53
Plce1	phospholipase C, epsilon 1	2.52
Rnf125	ring finger protein 125	2.51
Ifi35	interferon-induced protein 35	2.49
Parp3	poly (ADP-ribose) polymerase family, member 3	2.48
Fcer1g	Fc receptor, IgE, high affinity I, gamma polypeptide	2.48
Mfsd2	major facilitator superfamily domain containing 2	2.46
Il1rn	interleukin 1 receptor antagonist	2.46
Clec4a3	C-type lectin domain family 4, member a3	2.46
Pnpla2	patatin-like phospholipase domain containing 2	2.45
Gfap	glial fibrillary acidic protein	2.44
Rasip1	Ras interacting protein 1	2.44
Nudt6	nudix (nucleoside diphosphate linked moiety X)-type motif 6	2.44
Csf2rb	colony stimulating factor 2 receptor, beta, low-affinity (granulocyte-macrophage)	2.44
Hif3a	hypoxia inducible factor 3, alpha subunit	2.44
Atp2c2	ATPase, Ca++ transporting, type 2C, member 2	2.40
Mt1	metallothionein 1	2.39
Il1r2	interleukin 1 receptor, type II	2.38
Tcfcp2l1	transcription factor CP2-like 1	2.37
C1r	complement component 1, r subcomponent	2.36
Zfp36	zinc finger protein 36	2.35
Cd300lf	CD300 antigen like family member F	2.34
Msn	moesin	2.33
Arpc1b	actin related protein 2/3 complex, subunit 1B	2.31
Tlr7	toll-like receptor 7	2.31
Ddc	dopa decarboxylase	2.31
H2-L	histocompatibility 2, D region	2.31
Ncf1	neutrophil cytosolic factor 1	2.30
H2-D1	histocompatibility 2, D region locus 1	2.29

Anubl1	AN1, ubiquitin-like, homolog (Xenopus laevis)	2.28
C3ar1	complement component 3a receptor 1	2.27
Map3k8	mitogen-activated protein kinase kinase kinase 8	2.26
Ezr	ezrin	2.25
Tnfrsf1a	tumor necrosis factor receptor superfamily, member 1a	2.23
Parp14	poly (ADP-ribose) polymerase family, member 14	2.22
Cirbp	cold inducible RNA binding protein	2.21
Lgals9	lectin, galactose binding, soluble 9	2.21
H2-K1	histocompatibility 2, K1, K region	2.20
Cd274	CD274 antigen	2.18
Mgp	matrix Gla protein	2.18
Rbm3	RNA binding motif protein 3	2.18
Selp	selectin, platelet	2.17
Gpr182	G protein-coupled receptor 182	2.16
Procr	protein C receptor, endothelial	2.15
Angpt2	angiopoietin 2	2.14
Tmbim1	transmembrane BAX inhibitor motif containing 1	2.13
Tspo	translocator protein	2.12
Alox12b	arachidonate 12-lipoxygenase, 12R type	2.12
Parp9	poly (ADP-ribose) polymerase family, member 9	2.11
Tsc22d3	TSC22 domain family, member 3	2.10
Adm	adrenomedullin	2.10
Tmem176a	transmembrane protein 176A	2.10
Capg	capping protein (actin filament), gelsolin-like	2.10
Lyve1	lymphatic vessel endothelial hyaluronan receptor 1	2.07
Irf9	interferon regulatory factor 9	2.07
Vim	vimentin	2.07
Il13ra1	interleukin 13 receptor, alpha 1	2.07
Clic1	chloride intracellular channel 1	2.06
Hck	hemopoietic cell kinase	2.06
Cxcl9	chemokine (C-X-C motif) ligand 9	2.06
Akap12	A kinase (PRKA) anchor protein (gravin) 12	2.06
Synpo	synaptopodin	2.05
Rhou	ras homolog gene family, member U	2.05
Col5a3	collagen, type V, alpha 3	2.05
Ddit4	DNA-damage-inducible transcript 4	2.04
Anxa2	annexin A2	2.04
Psmb9	proteasome (prosome, macropain) subunit, beta type 9	2.03
Bcl3	B-cell leukemia/lymphoma 3	2.01
Pycard	PYD and CARD domain containing	2.01

Part B: List of all downregulated genes (FDR<0.05)

**Gene Symbol**	**Gene Title**	**FC**
Gpr34	G protein-coupled receptor 34	-7.85
P2ry12	purinergic receptor P2Y, G-protein coupled 12	-4.61
Serpinb1a	serine (or cysteine) peptidase inhibitor, clade B, member 1a	-3.75
Akr1c14	aldo-keto reductase family 1, member C14	-3.56
Itm2a	integral membrane protein 2A	-3.51
Cxcl12	chemokine (C-X-C motif) ligand 12	-3.45
Ugt8a	UDP galactosyltransferase 8A	-3.10
Tek	endothelial-specific receptor tyrosine kinase	-3.06
Slco1c1	solute carrier organic anion transporter family, member 1c1	-2.88
Slc40a1	solute carrier family 40 (iron-regulated transporter), member 1	-2.71
Myoc	myocilin	-2.66
Abcb1a	ATP-binding cassette, sub-family B (MDR/TAP), member 1A	-2.61
Slc22a8	solute carrier family 22 (organic anion transporter), member 8	-2.60
Fabp7	fatty acid binding protein 7, brain	-2.60
Pllp	plasma membrane proteolipid	-2.60
Aspa	aspartoacylase	-2.56
Pltp	phospholipid transfer protein	-2.52
Slc47a1	solute carrier family 47, member 1	-2.44
Ptprb	protein tyrosine phosphatase, receptor type, B	-2.44
Olfml3	olfactomedin-like 3	-2.39
Hist1h3b	histone cluster 1, H3b	-2.36
Gjb1	gap junction protein, beta 1	-2.34
Slc19a3	solute carrier family 19 (sodium/hydrogen exchanger), member 3	-2.33
Gjc2	gap junction protein, gamma 2	-2.31
Gulp1	GULP, engulfment adaptor PTB domain containing 1	-2.29
Hist1h4a	histone cluster 1, H4a	-2.24
Rlbp1	retinaldehyde binding protein 1	-2.21
Opalin	oligodendrocytic myelin paranodal and inner loop protein	-2.21
Tst	thiosulfate sulfurtransferase, mitochondrial	-2.20
Hist2h3c2	histone cluster 2, H3c2	-2.17
Rasgrp3	RAS, guanyl releasing protein 3	-2.14
Tnc	tenascin C	-2.07
Ranbp3l	RAN binding protein 3-like	-2.06
Ppp1r14a	protein phosphatase 1, regulatory (inhibitor) subunit 14A	-2.06
Gng11	guanine nucleotide binding protein (G protein), gamma 11	-2.06
Gldc	glycine decarboxylase	-2.05
Hist1h3a	histone cluster 1, H3a	-2.01

Supplementary Table 2. List of the genes in most significantly altered top ten canonical pathways.

**Ingenuity Canonical Pathways**	**-log (p-value)**	**Ratio**	**Genes**
Granulocyte adhesion and diapedesis	1.13E+01	1.07E-01	*C5ar1, Icam1, Mmp3, Tnfrsf1a, Ccl5, Sdc4, Csf3, Ccl9, Cxcl10, Il1r2, Selp, Ccl12, Il1rn, Ezr, Cxcl12, MMP12, CXCL2, Msn, Hspb1*
Agranulocyte adhesion and diapedesis	8.07E+00	8.47E-02	*C5ar1, Icam1, Mmp3, Tnfrsf1a, Sdc4, Ccl5, Ccl9, Cxcl10, Ccl12, Selp, Il1rn, Ezr, Cxcl12, MMP12, CXCL2, Msn*
Communication between innate and adaptive immune cells	6.85E+00	9.17E-02	*Cxcl10, Tlr2, Il1rn, HLA-B, Tlr7, Fcer1g, Cd86, Ccl5, HLA-E, Ccl9*
Acute phase response signaling	6.20E+00	7.78E-02	*C1r, Socs3, Serping1, Nfkbia, C3, Il1rn, Tnfrsf1a, Saa3, Saa1, Osmr, Serpina3n, Cp, LBP, A2m*
Dendritic cell maturation	5.85E+00	6.22E-02	*Tlr2, Col5a3, Nfkbia, Plce1, Icam1, Il1rn, Tnfrsf1a, FCGR2A, HLA-B, Fcer1g, Cd86, Fcgr2b, FCGR3A*
LXR/RXR activation	5.63E+00	8.09E-02	*Il1r2, C3, Msr1, Ccl12, Il1rn, Tnfrsf1a, Saa1, Cd14, LBP, PLTP, Apod*
Antigen presentation pathway	5.29E+00	1.50E-01	*Psmb9, NLRC5, HLA-B, Psmb8, Tap1, HLA-E*
Interferon signaling	5.09E+00	1.67E-01	*Ifit3, Ifitm1, Ifi35, Irf9, Psmb8, Tap1*
IL-10 signaling	4.84E+00	1.03E-01	*Il1r2, Socs3, Nfkbia, FCGR2A, Il1rn, Cd14, LBP, Fcgr2b*
Atherosclerosis signaling	4.76E+00	7.30E-02	*Alox12b, Col5a3, Icam1, Msr1, Ccl12, Mmp3, Selp, Il1rn, Pla2g3, Apod*

Ingenuity Pathway Analysis software (Ingenuity Systems) was used to analyze affected genes ($p<0.05$, FC>2) to identify the number of genes significantly associated with canonical pathways in the Ingenuity database. Top 10 pathways are presented.
